# The phosphate-solubilizing ability of *Penicillium guanacastense* and its effects on the growth of *Pinus massoniana* in phosphate-limiting conditions

**DOI:** 10.1242/bio.046797

**Published:** 2019-11-08

**Authors:** Huan Qiao, Xiao-Rui Sun, Xiao-Qin Wu, Gui-E. Li, Zao Wang, De-Wei Li

**Affiliations:** 1 Co-Innovation Center for Sustainable Forestry in Southern China, College of Forestry, Nanjing Forestry University, Nanjing, Jiangsu 210037, China; 2 The Connecticut Agricultural Experiment Station Valley Laboratory, Windsor, CT 06095, USA

**Keywords:** *Pinus massoniana*, Phosphate-solubilizing capability, Growth promotion, *Penicillium guanacastense*, Biological fertilizer

## Abstract

Microbes in soil can degrade insoluble inorganic and organic phosphorus, which are components of the soil phosphorus cycle and play an important role in plant growth. *Pinus massoniana* is a pioneer tree species used for afforestation in southern China and grows in poor, acidic soil. A shortage of available phosphorus in soil limits the growth of *P*. *massoniana*. To alleviate this situation, it is necessary to improve soil fertility. A fungal strain (JP-NJ2) with the ability to solubilize phosphate was isolated from the *P*. *massoniana* rhizosphere. The ability of JP-NJ2 to solubilize inorganic and organic phosphorus and promote the growth of *P*. *massoniana* was evaluated. It showed that JP-NJ2 could grow in NBRIP inorganic phosphate (AlPO_4_, FePO_4_·4H_2_O, and Ca_3_[PO_4_]_2_) fermentation broths, with the highest phosphorus concentration (1.93 mg/ml) and phosphate-solubilizing rate (43.7%) for AlPO_4_ and in Monkina organic phosphate fermentation broth with a phosphorus concentration of 0.153 mg/ml. The phosphate-solubilizing capability in inorganic and organic fermentation broths was negatively correlated with pH. JP-NJ2-produced acids at a total concentration of 4.7 g/l, which included gluconic (2.3 g/l), oxalic (1.1 g/l), lactic (0.7 g/l) and malonic (0.5 g/l) acids. It prioritized extracellular acidic phosphatase and combined with phytase to solubilize organic phosphates. The fungal suspension and extracellular metabolites from phosphate-solubilizing fungi promoted the shoot length of *P*. *massoniana* seedlings by 97.7% and 59.5%, respectively, while increasing the root crown diameter by 46.8% and 27.7%. JP-NJ2 was identified as *Penicillium guanacastense* based on its morphology and phylogenetic analyses of five genes/regions (ITS, *ben A*, *cmd*, *cox1* and *tef*). This is the first report on *P*. *guanacastense* isolated from pine tree rhizosphere soil in China and its high phosphate-solubilizing capability, which promoted the growth of *P*. *massoniana*. *P*. *guanacastense* JP-NJ2 has potential use as a biological fertilizer in forestry and farming.

## Abstract (precis)

**Summary:** Identification of a phosphate-solubilizing fungus and its first report in the application as a biological fertilizer.

## INTRODUCTION

Plant growth and development require many minerals, including phosphorus (P) (Vance, 2001; Cheng et al., 2018). In soils, unavailable inorganic P typically includes calcium phosphate (Ca-P), iron phosphate (Fe-P) and aluminum phosphate (Al-P) (Chuang et al., 2007; Wang and Cao, 2011). Plants have difficulty absorbing and utilizing the stable organic phosphorus that mainly exists in the form of phytate and other chelates in soil (Alam and Ladha, 2004). To alleviate the phosphorus shortage, chemical phosphate fertilizers are applied to soil, but more than 90% of soil phosphorus is not available to plants under natural conditions. Most of the applied phosphate fertilizers combine with metal ions in the soil to form insoluble phosphates (Hamdali et al., 2008). Consequently, the application of chemical fertilizer can fail to achieve the expected results and can lead to soil salinization, water eutrophication and ecological imbalance. Many studies have shown that soil microorganisms can dissolve insoluble phosphorus, which is not available to plants, and transform it into soluble phosphorus (Valverde et al., 2006; Goldstein, 2007; Singh and Reddy, 2011). Therefore, the use of microorganisms to improve soil fertility is a promising, renewable method.

Phosphate-solubilizing microbes in soil, including phosphate-solubilizing fungi (PSF) (Mehta et al., 2019) and bacteria (PSB) (Zheng et al., 2018), play an important role in phosphorus cycling. While the variety and quantity of PSB exceed those of PSF (Jin et al., 2006), the phosphate-solubilizing capability of fungi is generally superior to that of bacteria, and can be several dozen times higher. The genetic traits of fungi are more stable than those of bacteria. The phosphate-solubilizing capability of bacteria declines or is lost during subculture (Leyval et al., 1993; Vassilev et al., 1995). PSF can be applied to a variety of crops in different ecosystems. For example, *Aspergillus niger* and *Penicillium chrysogenum* promote the growth and nutrient uptake of groundnut (*Arachis hypogaea* L.) (Jitendra et al., 2011). Inoculation with the PSF *Aspergillus niger* significantly increases the growth, root nodulation and yield of soybean plants (Saxena et al., 2016). Nevertheless, the effects of PSF on phosphate solubility and growth in forest ecosystems are poorly understood.

Masson pine (*Pinus massoniana* Lamb.) is a pioneer tree species used for afforestation in southern China (Xu and Xiao, 2017). Masson pine stands are found mainly in the tropical and subtropical regions, where red soil is widely distributed, and the available P can be bound easily by calcium (Ca), aluminum (Al) and iron (Fe) through chemical precipitation or physical adsorption (Zhou et al., 2005; Wu et al., 2011). Highly weathered and acidiﬁed red soil with low P availability is dominant in the area of artificial afforestation and is thus one of the most important factors causing a decline in the productivity of Masson pine stands (Zhou et al., 2005; Zhang et al., 2010, 2013). To ameliorate this, it is necessary to improve soil fertility. This study isolated a fungal strain with P-solubilizing ability from the rhizosphere soil of *P.* *massoniana* in China, and investigated its taxonomic status morphologically and using multi-locus phylogenetic analyses (ITS, *ben A*, *cmd*, *cox1* and *tef*). The degradation of insoluble phosphate and the secretion of organic acids, phosphatase and phytase by this strain were investigated. In addition, its effects on the growth of *P*. *massoniana* were evaluated.

## RESULTS

### Selection for the efficient PSF

**Fig. 1.**
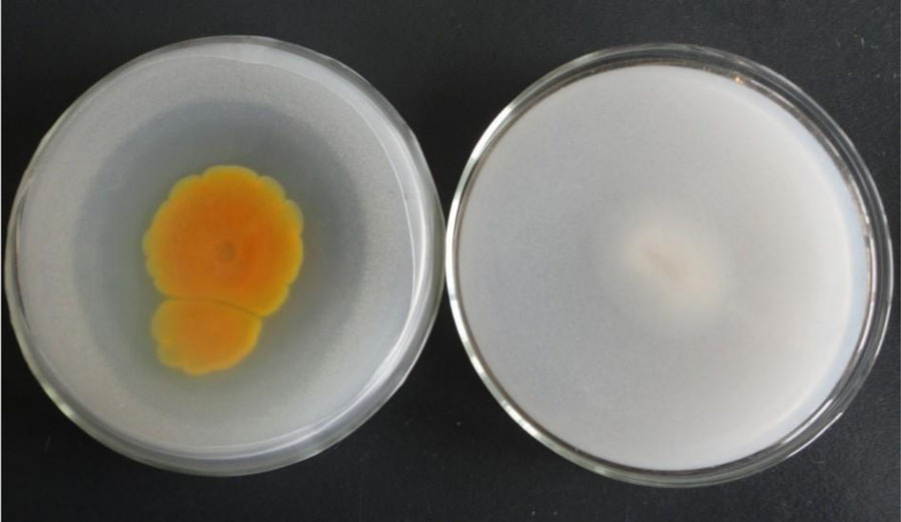
**Circular band of solubilization by JP-NJ2 strain on (left) NBRIP and (right) PDA.**

Eighteen PSF strains were obtained from 30 Masson pine rhizosphere soil samples. We measured the diameter of the colony (*D*) and circular band of dissolved phosphorus (*d*) and the ratio *d*/*D* was calculated. Of these, 14 strains had *d*/*D*>0.5 and one strain with an obvious circular band of dissolved phosphorus had *d*/*D*>1. The latter strain was named JP-NJ2 (Fig. 1). The pH of rhizosphere soil was 5.8.

### Types and content of organic acids

The generation of organic acids is an important mechanism enabling the dissolution of phosphate by PSF (Sperber, 1957; Louw and Webley, 1959; Xi et al., 2007).The total concentration of acids produced by JP-NJ2 was 4.7 g/l and the acids included gluconic (2.3 g/l), oxalic (1.1 g/l), lactic (0.7 g/l) and malonic (0.5 g/l) acids in order.

### Dynamic changes in the inorganic phosphate-solubilizing capability

**Fig. 2.**
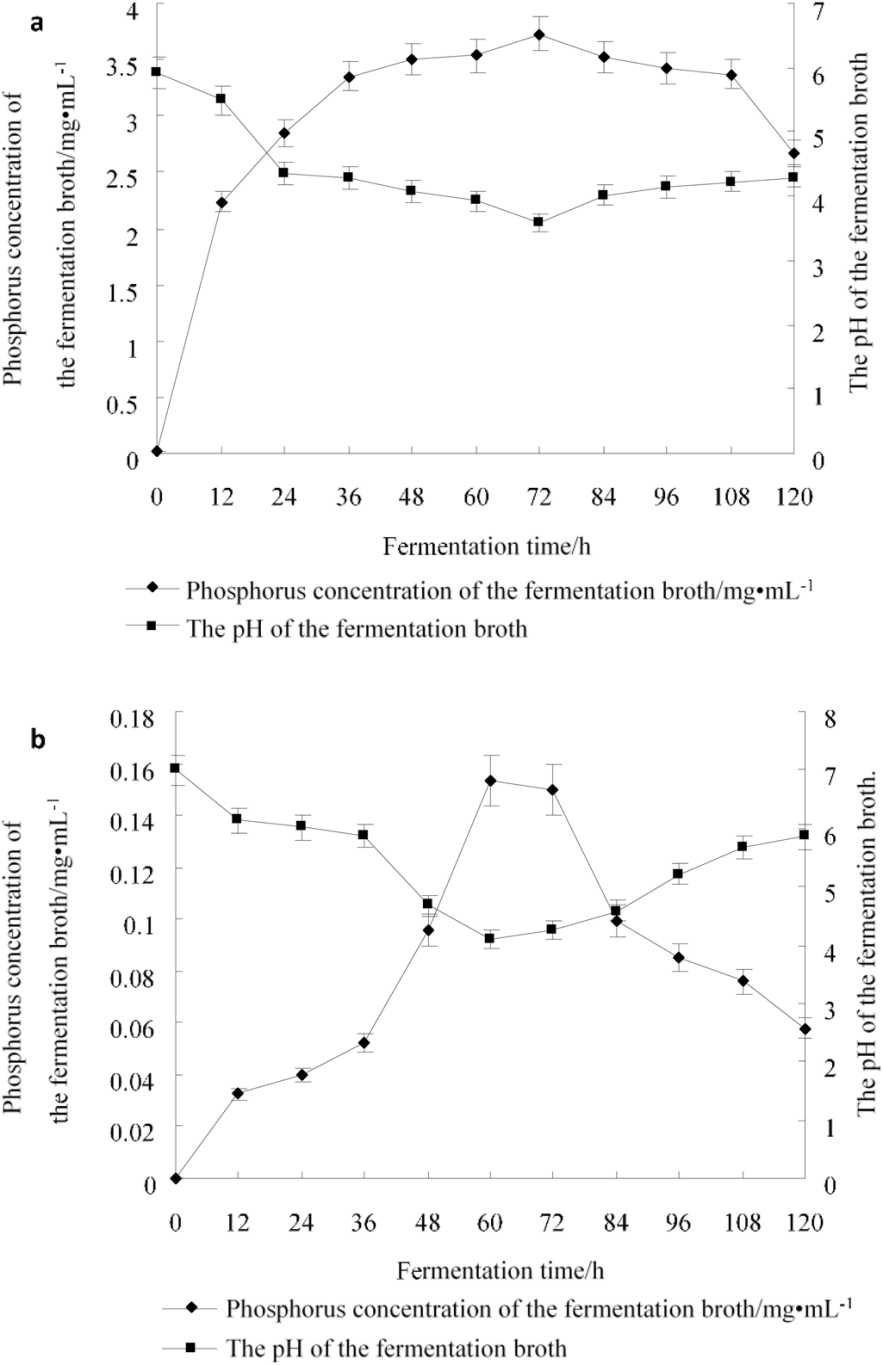
**pH and phosphorus concentration changes.** Changes in pH and phosphorus concentration of JP-NJ2 cultured in NBRIP inorganic fermentation broth (A) and in Monkina organic fermentation broth (B) over 120 h. The error bars show standard deviation. Every treatment included three biological replicates (*n*=3), each of which contained three technical replicates.

The phosphate solubilized by JP-NJ2 increased gradually over 60 h and reached the highest phosphorus concentration (3.7 g/l) at 72 h. The soluble phosphorus content and pH were negatively correlated and the pH remained low after 72 h (Fig. 2A).

### Phosphate-solubilizing capability of PSF in different inorganic phosphorus compounds

**Fig. 3.**
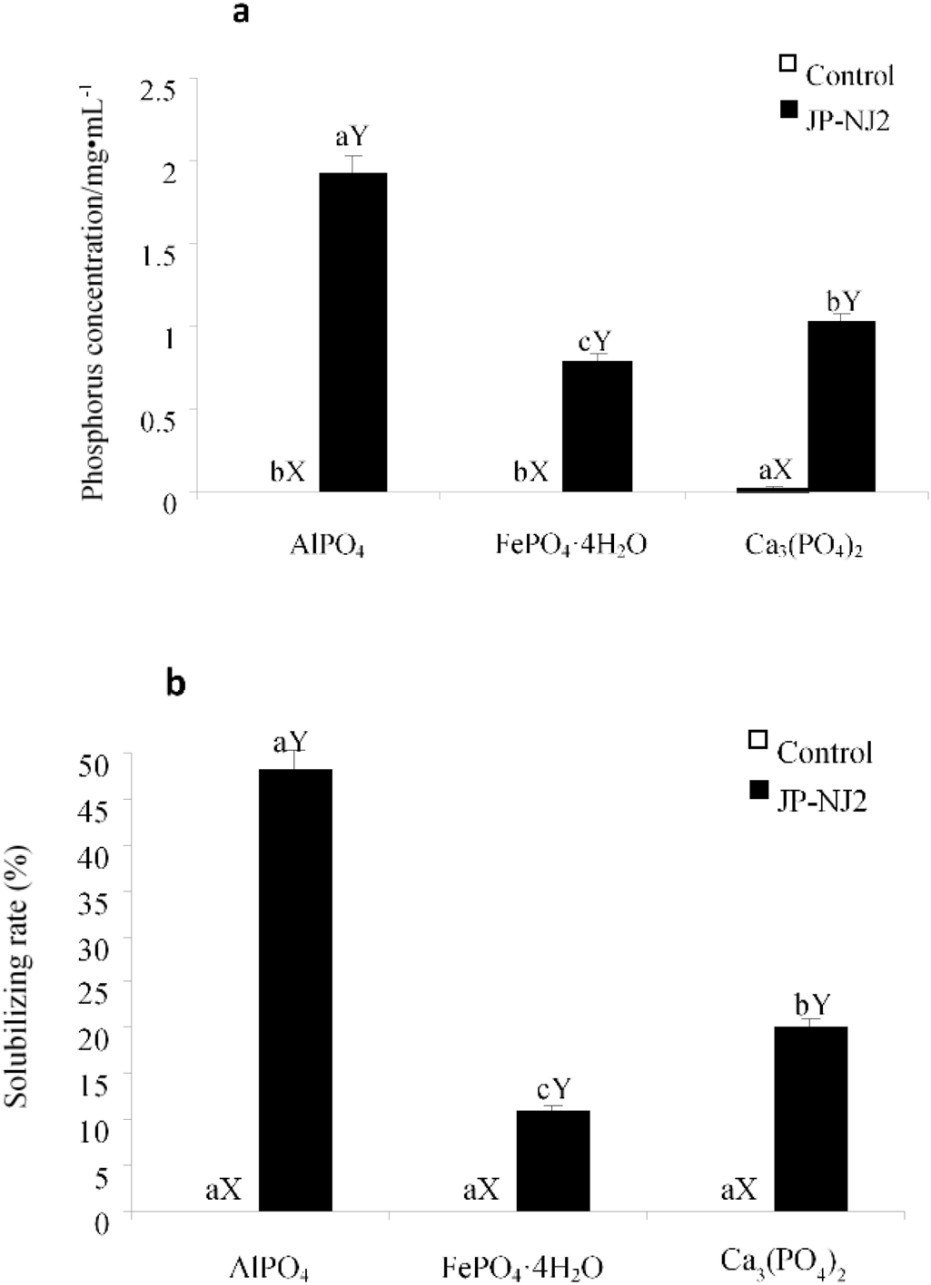
**JP-NJ2 solubilization rates.** Solubilization of different inorganic insoluble phosphates (A) and phosphate-solubilizing rate (B) by JP-NJ2 (*P*<0.05). The letters (both capital and lowercase) by the bars mean the control group (X), JP-NJ2 group (Y) and 5% significance of difference (a, b, c). The error bars show standard deviation. Every treatment included six biological replicates (*n*=6), each of which contained three technical replicates.

JP-NJ2 grew in media containing three different inorganic phosphates. However, the efficiency with which different phosphorus compounds were dissolved differed significantly (*P*<0.05) (Fig. 3A). The strain had the highest phosphate-solubilizing capability in medium containing AlPO_4_, followed in order by Ca_3_(PO_4_)_2_ and FePO_4_·4H_2_O. The phosphorus concentration reached 1.93 mg/ml and the phosphate-solubilizing rate was 43.7% in medium containing AlPO_4_ and 16.9%, 8.5% and 0.6% in other two media and the control, respectively (Fig. 3A,B). The differences were significant (all *P*<0.05) (Fig. 3B).

### Dynamic changes in the organic phosphate-solubilizing capability

When fermented at 25°C for 5 days, the phosphate-solubilizing capability of the strain increased gradually until 48 h, peaked at 60 h (0.153 mg/ml), and declined afterwards. The phosphate-solubilizing concentration and pH were negatively correlated and the pH was lowest (4.1) at 60 h and increased subsequently (Fig. 2B).

### Phosphatase and phytase activities

Phosphatase and phytase are the key for transforming organic phosphates into dissolvable phosphorus (Liang et al., 2011). After culture in potato dextrose broth (PDB) at 25°C for 4–5 days, the JP-NJ2 had high phosphatase activity and extracellular phosphatase predominated (*P*<0.01) (Table 1). The activity of extracellular acid phosphatase produced by the strain was significantly (*P*<0.01) higher than that of intracellular acid phosphatase. There were no significant differences in the activities of intra- and extracellular alkaline phosphatase. The extracellular acid phosphatase activity of JP-NJ2 influenced its ability to dissolve lecithin. JP-NJ2 also showed phytase activity (Table 1), which would affect its ability to dissolve phytate.

**Table 1.**
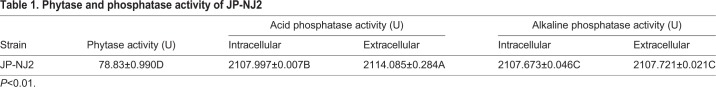
**Phytase and phosphatase activity of JP-NJ2**

### Effects of JP-NJ2 on the growth of Masson pine

The growth-promoting ability of the highly efficient phosphate-solubilizing fungus JP-NJ2 on *P. massoniana* was determined. An inoculation test was divided into four treatments, each of which was treated with 15 ml of one of the following corresponding substances: (A) fungal suspension; (B) extracellular metabolites produced by the test strain; (C) blank culture medium (PDB) or (D) sterile saline (control). Each treatment was repeated 20 times and the inoculated pine seedlings were placed in a greenhouse (20°C) with unified management and timely watering. Fungal suspension and extracellular metabolites produced by the test strain greatly improved the growth of Masson pine (Fig. S1). The shoot length and root crown diameter of the Masson pine seedlings were measured after 450 days. Both the fungal suspension and extracellular metabolites from PSF JP-NJ2 promoted the shoot length of Masson pine, resulting in increments of 97.7% (*P*<0.05) and 59.5% (*P*<0.05), respectively, while the root crown diameter increased by 46.8% (*P*<0.05) and 27.7% (*P*>0.05) (Table 2).

**Table 2.**
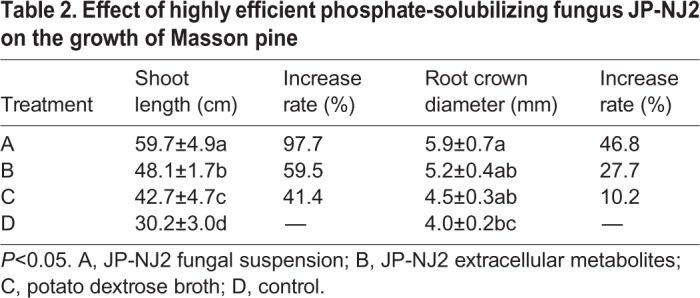
**Effect of highly efficient phosphate-solubilizing fungus JP-NJ2 on the growth of Masson pine**

### Morphological identification of the efficient PSF strain, JP-NJ2

**Fig. 4.**
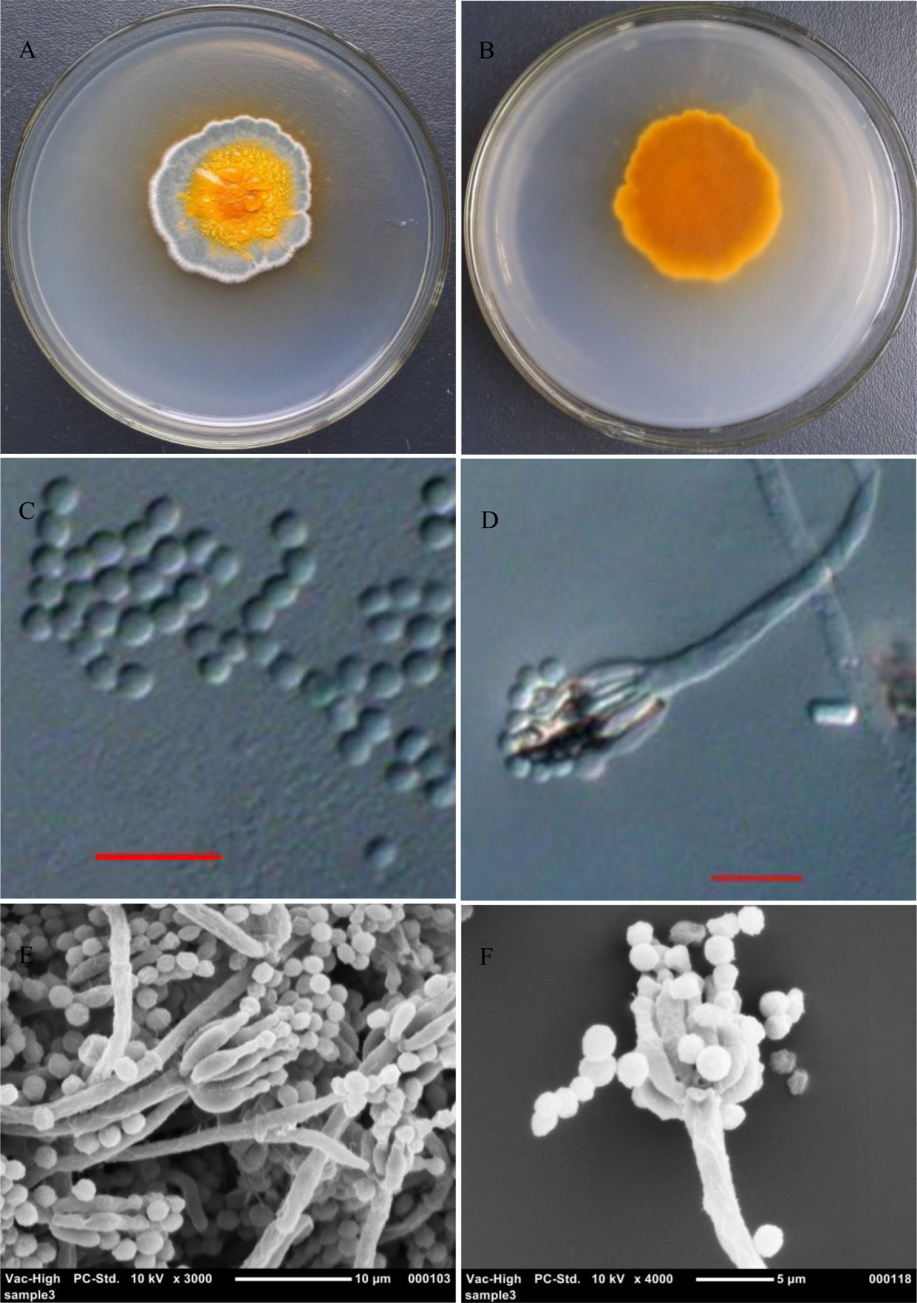
**Morphological characteristics of JP-NJ2.** Light microscopy (A) colony on CYA; (B) colony reverse on CYA; (C) conidia; (D) conidiophore. Scanning electron microscopy of conidiophores (E,F). Scale bars: (C–E) 10 μm; (F) 5 μm.

Colonies of the JP-NJ2 strain reached 22–34 mm in diameter on CYA medium at 25°C in 7 days and were almost flat, sometimes with regular wrinkles. The texture was flocculent and velvet with six to eight grooves (Fig. 4). The conidial surface of the colonies had three concentric rings: a central orange ring, a green-bluish gray middle ring and a white outmost ring (2–5 mm). The strain produced a moderate clear exudate and soluble yellow or orange-yellow pigments after 5–7 days (Fig. 4A). The reverse side of the colony was orange (Fig. 4B).

The fungal hyphae were colorless, translucent and 2–3 μm wide. The conidiophores were sparse, septate, roughed, monoverticillate, 83–101×2.2–3.1 μm, unbranched or with very few primary branches, and had a terminal vesicle 3.9–5.3 μm wide. The metulae were in terminal whorls of two to six. The phialides were colorless, smooth, 7.8–12×1.9–3.3 μm and tapering to a narrow conidium-bearing tube (Fig. 4D–F). The conidia were one-celled, catenate, colorless, globose to subglobose, slightly roughed and 2.3–2.9 μm in diameter (Fig. 4C).

Based on its morphological characters, JP-NJ2 was identified as *Penicillium*.

### Identification of the efficient PSF strain, JP-NJ2, based on phylogenetic analyses

**Fig. 5.**
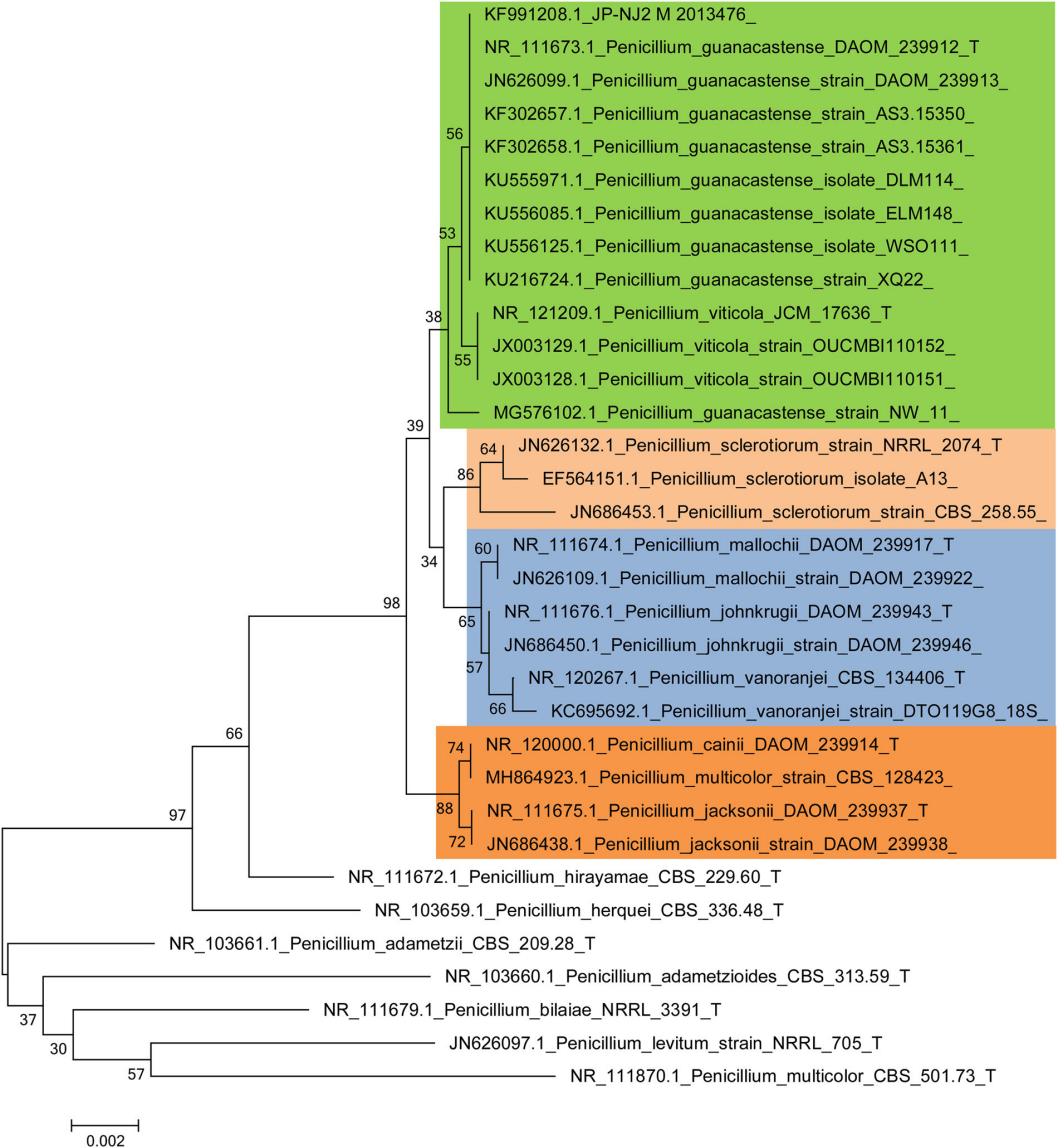
**Neighbor-joining tree of phosphate-solubilizing fungus JP-NJ2 based on ITS sequences.** Bootstrap values on 1000 replications are shown at nodes of the tree. Scale bar: 0.002 substitutions per nucleotide position. T indicates ex type (ex, type strain).

**Fig. 6.**
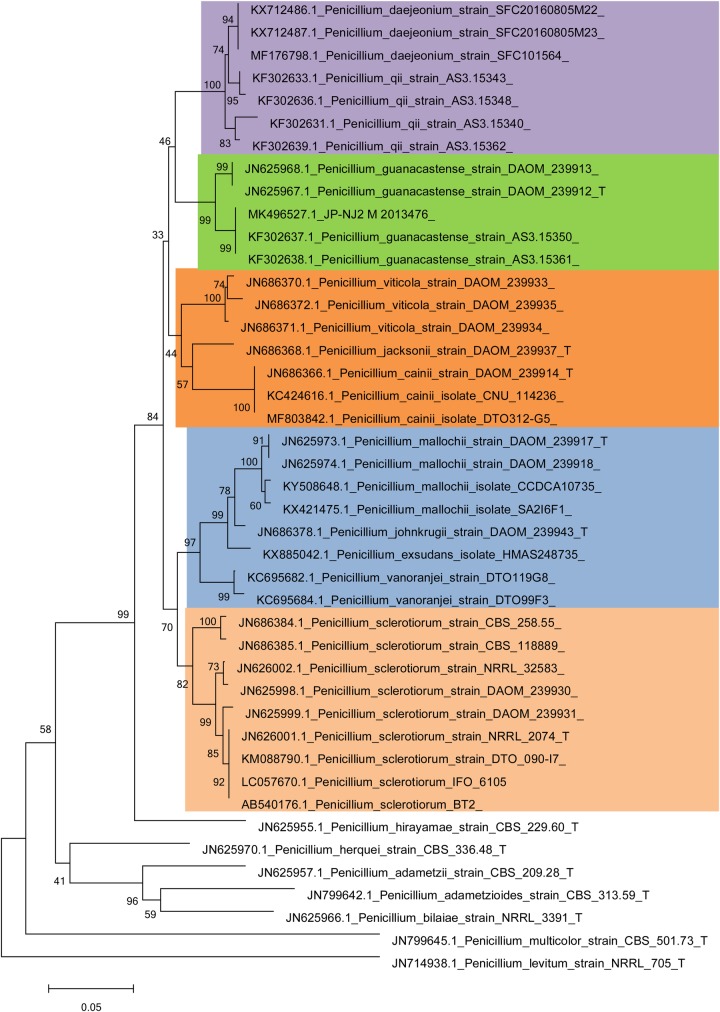
**Neighbor-joining tree of phosphate-solubilizing fungus JP-NJ2 based on *ben A* sequences.** Bootstrap values on 1000 replications are shown at nodes of the tree. Scale bar: 0.05 substitutions per nucleotide position. T indicates ex type.

Figs 5 and 6 and Figs S2, S3 and S4 show the phylogenetic relationships between the JP-NJ2 strain and representative fungal strains in neighbor-joining trees constructed using five barcode markers. The ITS sequence of the JP-NJ2 strain clustered with eight other strains of *Penicillium* *guanacastense* (bootstrap=56%). The *ben A* sequence of the JP-NJ2 strain clustered with four other strains of *P*. *guanacastense* (bootstrap=99%). The *cmd* sequence of the JP-NJ2 clustered perfectly with four other strains of *P*. *guanacastense* (bootstrap=100%). The *cox1* sequence of the JP-NJ2 strain clustered with two other strains of *P*. *guanacastense* (bootstrap=94%). The *tef* sequence of JP-NJ2 clustered with two other strains of *P*. *guanacastense* (bootstrap=81%). All of the sequences of the DNA barcode markers of the JP-NJ2 strain clustered with the corresponding *P*. *guanacastense* sequences.

Among the phylogenetic trees of the DNA genes/region, that of ITS was less clearly resolved and although most species formed monophyletic groups in the strict consensus trees, several had low bootstrap support. One (*P*. *guanacastense* strain NW-11) of the ten *P*. *guanacastense* sequences did not group with the *P*. *guanacastense* type strain (*P*. *guanacastense* DAOM_239912_T) and had low bootstrap support (bootstrap=38%) (Fig. 5). *Ben* *A* can successfully be used for accurately identifying *Penicillium* species (Visagie et al., 2014). In the trees constructed using the other DNA barcode markers (Fig. 6; Figs S2 and S4), the JP-NJ2 strain clustered in the green area with the other *P*. *guanacastense*, *Penicillium* *sclerotiorum* was in the pink area, *Penicillium* *jacksonii* and *Penicillium* *cainii* were in the orange area, and *Penicillium johnkrugii* and *Penicillium mallochii* clustered in the blue area. A 648 bp segment of mitochondrial cytochrome c oxidase 1 (*cox1*) is the core barcode region for animals and its utility has been confirmed in fungi (Seifert et al., 2007). Unlike the other four genes, the *cox1* phylogenetic tree showed diversity (Fig. S3), *P*. *guanacastense* JP-NJ2 produced unique clusters due to the specificity of *cox1* with *P*. *jacksonii*, *P. cainii* and *Penicillium* *viticola*. These clusters are similar to the results of Rivera and Seifert (2011). Combined with the morphological observations, this indicates that the JP-NJ2 strain is *P*. *guanacastense*. *P*. *guanacastense* JP-NJ2 (M 2013476) was deposited in the China Center for Type Culture Collection (CCTCC).

## DISCUSSION

PSF are an important group of rhizosphere microbes (Valverde et al., 2006; Singh and Reddy, 2011). The fungal strain JP-NJ2, which had high phosphate-solubilizing capability, was isolated from the rhizosphere soil of Masson pine stands and identified as *P.* *guanacastense* using morphological characters and phylogenetic analyses of five genes/region (ITS, *ben A*, *cmd*, *cox1* and *tef*). Rivera et al. (2012) first described *P*. *guanacastense* as a new species after studies led to the realization that *P.* *sclerotiorum* included a species complex that was similar morphologically, but included distinct species according to phylogenetic analyses (Rivera and Seifert, 2011). *P*. *guanacastense* resembles *P*. *sclerotiorum* phylogenetically and morphologically. However, it does not produce sclerotia and differs subtly in conidial shape. The conidia of *P*. *guanacastense* are globose to subglobose while those of *P*. *sclerotiorum* are ellipsoid (Kong, 2007; Rivera et al., 2012). In our study, colonies of *P.* *jacksonii* were similar to those of *P*. *guanacastense* JP-NJ2 on CYA: 10% of the JP-NJ2 conidiophores branched, while 50% of the *P*. *jacksonii* conidiophores branched and their green conidia were the darkest within *Penicillium*. In addition, more vesiculate conidiophore apices are observed in *P*. *guanacastense* than in *P.* *viticola* (Nonaka et al., 2011). The morphology of colonies of the PSF JP-NJ2 and *P*. *guanacastense* did not differ in CYA medium, but differed from those of *P*. *sclerotiorum* and *P*. *jacksonii*. The conidia of JP-NJ2 were globose to subglobose and colorless (Fig. 4C). These results confirm that JP-NJ2 is *P*. *guanacastense*.

We conducted a blast search using all of the DNA sequences from the JP-NJ2 strain (from NCBI). This indicated that most previously submitted *P*. *guanacastense* sequences were ITS sequences, most of which had been submitted without related publications. In addition, the publications on *P*. *guanacastense* focus on its taxonomic status and phylogenetics, and do not explore function (Rivera and Seifert, 2011; Rivera et al., 2012). The first report on *P*. *guanacastense* isolated from the gut of *Eutelia* sp. (Noctuidae), reared on foliage of *Spondias mombin* (Anacardiaceae) in Costa Rica was by Rivera and Seifert (2011). We have summarized all of the known sources of *P*. *guanacastense*, including the guts of caterpillars of *Eutelia* sp. (Rivera et al., 2012), roots of a coastal plant, cashew nuts and *Euphausia superba* (sequences deposited in GenBank, Table S1; related papers unpublished). Our *P*. *guanacastense* JP-NJ2 was isolated from the rhizosphere soil of the Masson pine. Therefore, *P*. *guanacastense* might have a wide range of hosts or substrates and be present in forest soil.

Phosphate solubilization is a well-known phenomenon in *Penicillium*. The most famous example is *Penicillium bilaiae*, which is sold commercially by NovoZymes as a soil inoculant. Efficacy and comprehensive data on compatibility with seed-applied pesticides were confirmed by extensive field research to ensure the interests of farmers (Leggett et al., 2007). *Penicillium bilaiae* also can be used as a P-solubilizing microbe to solubilize P from thermally converted sewage sludge (Raymond et al., 2018), but the approach has to be further investigated regarding its effects in a soil/plant system. Further research has investigated several *Penicillium* species, notably *P. radicum*, *P. bilaiae* (strain RS7B-SD1), and an unidentified *Penicillium* sp. designated strain KC6-W2; for example the comparison by Wakelin et al. (2007). These *Penicillium* species increase the growth and P nutrition of herbaceous plants (wheat, medic and lentil) in three soils of neutral to alkaline pH reaction. The strongest plant growth promoting (PGP) strain was *Penicillium* sp. KC6-W2, which stimulated significant increases in shoot growth and dry mass. Levels of PGP by these *Penicillium* species were associated with increased uptake of P to the shoot to some extent. In our study, we set our sights toward the phosphate-solubilizing characteristic of *P. guanacastense* and its growth-promoting ability on a ligneous plant (Masson pine). Among all five phylogenetic trees, *P. guanacastense* forms a separate branch in the genus *Penicillium* and is distinctly not closely related to *P. bilaiae*; it is also certainly remotely related to *P. radicum* as its taxonomic status*,* and *P. radicum* was classified as the anamorph of *Talaromyces radicus* (Samson et al., 2011). It may give further proof of the existence of widely distributed phosphate solubilization in *Penicillium*. Although this is really a common case in *Penicillium*, it differs in phosphate solubilization capacity among *Penicillium* species due to the different secretion of organic acid, phosphatase and phytase or the other involved phosphate-solubilizing mechanisms.

The phosphate-solubilizing ability of microbes is correlated with the structures and components of different insoluble phosphorus compounds (Chuang et al., 2007). The phosphate-solubilizing efficiency of different PSF strains/species varies markedly (Reddy et al., 2002). For example, *Aspergillus tubingensis* and *A*. *niger* have different phosphate-solubilizing capabilities. *Paecilomyces marquandii* from different origins can utilize amylase and has different phosphate-solubilizing capabilities under various nitrogen sources (Ahuja et al., 2007). The effects of applying phosphate-solubilizing microbial fertilizer are often associated with soil characteristics. In China, Masson pine mainly grows in acidic soils south of the Yangtze River. Under strong acidic conditions, aluminum in the clay mineral alumina layer is released into the surface of soil colloid and releases exchangeable aluminum ions. Therefore, AlPO_4_ is the major insoluble phosphorus compound in soils in southern China (Xu et al., 2012). In our experiments, *P*. *guanacastense* JP-NJ2 grew in medium containing one of three different phosphorus compounds. It dissolved AlPO_4_ best (phosphate-solubilizing value 1.93 mg/ml), followed by Ca_3_(PO_4_)_2_. Therefore, phosphorus compounds have significant effects on the inorganic phosphate-solubilizing capability of *P*. *guanacastense* JP-NJ2.

Microbes can break down rock phosphate, and interactions among microbes that release organic acids and other products onto the surfaces of minerals may liberate ions from their surface layers (Reyes et al., 2006). The types and amounts of organic acids influence the anion-restraining ability of cations adsorbed by phosphate radicals and organic acids combined with metal ions such as Al^3+^, Ca^2+^ and Fe^3+^ (Chen et al., 2004). Hameeda et al. (2006) found that the production of gluconic acid increases phosphate-solubilizing capability. Elbahrany (1995) believed that oxalic acid was the main mechanism causing the dissolution of phosphate. In our study, the gluconic acid concentration was the highest (2.3 g/l), followed by oxalic acid (1.1 g/l), suggesting that both played an important role in the dissolution of AlPO_4_ and Ca_3_(PO_4_)_2_ by *P*. *guanacastense* JP-NJ2.

Phosphate-solubilizing microbes mainly secrete phosphatase for enzymolysis to decompose organic phosphate (Wallander, 2000). PSF can secrete phytase and phosphatase to transform organic phosphates into dissolvable phosphorus under low phosphorus stress (Lu, 2002; Liang et al., 2011). In our study, *P*. *guanacastense* JP-NJ2 has phosphatase high-activity, which had a dominant role in its phosphate-solubilizing action. The degradation of lecithin depends on the role of acid phosphatase (Mehra et al., 2017), and alkaline phosphatase activity has a great effect on the degradation of organophosphorus pesticides (Chu et al., 2018). In addition, *P*. *guanacastense* JP-NJ2 has phytase activity, which also plays a role in the decomposition of phytate.

Compared to the soluble phosphorus circular band determination method, determining the phosphate concentration in liquid medium is more reliable. Considering the phosphate-solubilizing efficiency of *P*. *guanacastense* JP-NJ2, we determined its phosphate concentration under fermentation conditions. This also demonstrated that *P*. *guanacastense* JP-NJ2 has a high capability to solubilize inorganic phosphorus. Its capacity for solubilizing Ca_3_(PO_4_)_2_ was 3.7 g/l at 72 h (Fig. 2A). JP-NJ2 dissolved more lecithin (0.153 g/l) (Fig. 2B) than that reported for *Burkholderia arboris* N85 (0.03 g/l) obtained from *Cymbidium goeringii* roots (Xu et al., 2013). This suggests that *P*. *guanacastense* JP-NJ2 could be applied in the field in the future.

In summary, phosphate-solubilizing microbial fertilizer can increase the efficiency of phosphorus uptake in the soil and improve plant production (Peix et al., 2001; Zaidi et al., 2003). Treatments using fungal suspension and extracellular metabolites from the JP-NJ2 strain promoted the shoot lengths of Masson pine seedlings by 97.7% (*P*<0.05) and 59.5% (*P*<0.05), respectively, while root crown diameters increased by 46.8% (*P*<0.05) and 27.7% (*P*>0.05). The growth promotion was greater with the fungal suspension treatment than with the extracellular metabolite treatment. This suggests that the growth-promoting effect is due to metabolites secreted during the vital biological processes of PSF. The *P*. *guanacastense* JP-NJ2 evaluated in this study solubilized phosphate and promoted the growth of *P*. *massoniana*. Therefore, it might be used to improve soil fertility in nurseries and forestry practice. Because the activities of *P*. *guanacastense* were determined in the laboratory and greenhouse, these results should be verified in the field. Before this, safety tests of this fungus should be conducted to ensure that it does not pose any risks to the public, animals or plants.

## MATERIALS AND METHODS

### Soil samples

Soil samples were collected from a 50-year-old Masson pine forest in Jiangsu Academy of Forestry, China, on 5 September 2011. The samples were collected by digging 20–30 cm in depth after removing the topsoil within half a meter of the rhizosphere of 10 Masson pine trees. Rootlets and the adherent soil samples from Masson pine roots were placed in sterile polyethylene bags and stored at 4°C. The Masson pines selected were 20 m apart from each other. Three replicates were obtained from each Masson pine. The pH of rhizosphere soil was determined using a glass electrode and with a soil-to-deionized water ratio of 1:2 (Heineman et al., 2016).

### Isolation of PSF

First, 10 g adherent soil brushed off Masson pine rootlets and 90 ml sterile distilled water were placed in a flask, to which 10 glass beads of 4 mm diameter were added. The suspension was oscillated at 180 rpm for 30 min at room temperature. Then the supernatant was allowed to sit for 10 min before being diluted to 10^−2^, 10^−3^, 10^−4^, 10^−5^ and 10^−6^. Next, 100 µl 10^−4^, 10^−5^ and 10^−6^ dilution suspension was spread on three inorganic National Botanical Research Institute phosphate (NBRIP) medium plates (Shekhar, 1999) containing dextrose 10 g, (NH_4_)_2_SO_4_ 0.1 g, MgSO_4_·7H_2_O 0.25 g, MgCl_2_·6H_2_O 5.0 g, Ca_3_ (PO_4_)_2_ 5.0 g and dd H_2_O 1000 ml, pH 6.8–7.2 and incubated at 25°C for 10 days. The reagents used in the medium were purchased from (Beijing Chemical Co., analytical grade). Colonies showing a distinct circular band of dissolved phosphorus were selected. The diameter of the colony (*D*) and circular band of dissolved phosphorus (*d*) were measured and the ratio *d*/*D* was calculated. The phosphate-solubilizing capability was categorized based on the *d*/*D* ratio: high, *d*/*D*>1; medium, 0.5<*d*/*D*<1; and low, *d*/*D*<0.5. The fungal isolates were purified and cultivated in potato dextrose agar (PDA) slant cultures at 25°C for 7–10 days. The fully developed slants were stored at 4°C.

### Organic acids in the PSF strain

The organic acids produced by the strain tested were identified and quantified using high-performance liquid chromatography (Agilent 1200 liquid chromatograph, Santa Clara, USA) after the strain was cultured in 50 ml NBRIP liquid medium on a shaker at 25°C for 4–5 days (Xi et al., 2007).

Gluconic acid was measured using an Agilent ZORBAX SB-Phenyl chromatography column (4.6×250 mm, 5 μm). The mobile phase consisted of (A) 0.1% H_3_PO_4_ and (B) CH_3_CN at a flow rate of 1 ml/min. The gradient was 40% B phase at 0–4 min and 90% B phase at 5–7 min. The injection volume was 2 μl and the determination wavelength 203 nm (Zeng et al., 2016). Other organic acids were measured in a Thermo Hypersil Gold column (250×4.6 mm, 5 μm); mobile phase, (A) 50 mmol/l KH_2_PO_4_, pH 2.5 and (B) methanol; flow rate, 1 ml/min; gradient, 0–8 min 0% B phase, 8–13 min 40% B phase; determination wavelength 203 nm; injection volume 2 μl.

### Inorganic phosphate-solubilizing capability of the PSF strain

The soluble phosphorus content was determined in fermentation broth by phosphorus molybdenum blue spectrophotometry. The PSF strain tested was inoculated on PDB and cultured on a shaker (180 r min^−1^) at 25°C for 4 days. Then, 150 ml JP-NJ2 stock solution was placed in a fermenter (Shanghai Guo Qiang) containing 1.5 l NBRIP medium and fermented at 25°C, 300 rpm, pH 7.2, 0.8 VVM. The soluble phosphorus content in the fermentation broth was measured at 12 h intervals for 120 h by spectrophotometry (Zeng et al., 2016). Every treatment included three biological replicates (three separate fermentation cultures) (*n*=3), each of which contained three technical replicates (three data measurements).

Three different NBRIP liquid mediums were prepared containing one phosphorus compound each from AlPO_4_, FePO_4_·4H_2_O and Ca_3_(PO_4_)_2_ added based on the same phosphorus content (1 g/l of NBRIP medium). The PSF strain was inoculated onto each medium and cultured on a shaker for 7 days before determining the soluble phosphorus contents in the fermentation broths. The phosphate-solubilizing rate was calculated using the following formula: ([soluble phosphorus content of inoculation group – soluble phosphorus content of control group]/[inorganic phosphorus content of each experimental group])×100. Every treatment included six biological replicates (six separate cultures) (*n*=3), each of which contained three technical replicates (three data measurements).

### Organic phosphate-solubilizing capability of the PSF strain

The fungal strain liquid (150 ml) was poured into a fermenter (Shanghai Guo Qiang) containing 1.5 l of Monkina medium (Feng et al., 2003) and fermented at 25°C, pH 7.2. Monkina medium contains: sucrose 10 g, (NH_4_)_2_SO_4_ 0.5 g, MgSO_4_·7H_2_O 0.3 g, NaCl 0.3 g, KCl 0.3 g, FeSO_4_·7H_2_O 0.03 g, MnSO_4_·7H_2_O 0.03 g, lecithin 0.6 g, CaCO_3_ 5 g and dd H_2_O 1000 ml, pH 7.2. The soluble phosphorus concentrations in the fermentation broths were measured every 12 h for 120 h. Lecithin is sourced from Shanghai Organic Chemical Co. and other reagents in the medium were purchased from Beijing Chemical Co. analytical grade. Every treatment included three biological replicates (three separate fermentation cultures) (*n*=3), each of which contained three technical replicates (three data measurements).

The phosphatase activity of the strain was determined by colorimetry using MUB reagent containing trimethylaminomethane 12.1 g, maleic acid 11.6 g, citric acid 14.0 g, boric acid 6.3 g in 488 ml 1 mol/l NaOH solution, diluted to 1000 ml with dd H_2_O, and then mixed. MUB reagent at pH 6.5 was used to determine acid phosphatase and MUB reagent at pH 11 was used to determine alkaline phosphatase at 420 nm after the stain was cultured in PDB on a shaker at 25°C for 4–5 days. The units of phosphatase activity (U) were defined as the amount of phosphatase required to produce 1 μmol inorganic phosphate in 1 min (Hofmann et al., 2016). Phytase activity assay was as follows: the fungal strain was inoculated on PDB medium and the suspension was oscillated at 180 rpm at 25°C for 4–5 days and centrifuged at 10,000 rpm for 10 min. The supernatant was decanted into a 50 ml centrifuge tube. The reaction mixture consisted of 1 ml acetate buffer (0.2 M, pH 5.5, containing 1 mM sodium phytate) and 2 ml culture supernatant (2 ml autoclaved non-inoculated medium was used as a control). The reaction was terminated by adding 1 ml 10% trichloroacetic acid after 30 min at 37°C. The absorbance was measured at 415 nm by spectrophotometry. Similarly, the unit of phytase activity (U) was defined as the amount of phytase required to produce inorganic phosphates from sodium phytate solution per minute (Makoudi et al., 2018). Every treatment included six biological replicates (six separate cultures) (*n*=6), each of which contained three technical replicates (three data measurements).

### Seed treatment and inoculation of the PSF strain on *P. massoniana* seedlings

The PSF strain was cultured in PDB medium, oscillated (180 rpm) at 25°C for 7 days, and then centrifuged in a centrifuge tube for 5 min (4°C, 6000 rpm). Part of the supernatant liquid was extracellular metabolites produced by the test strain, and the bottom was the part of mycelia (including conidia). The mycelium pellets at the bottom of the centrifuge tube were transferred to a commercial blender to be fractured and then diluted in a series of concentrations for subsequent microscopic count. The container above this kind of commercial blender can be taken down to be sterilized by high-pressure steam. The machine can also be used to fracture plant leaves. The fractured mycelium pellets and attached conidia were diluted with sterile saline to prepare treatment group A; fungal suspensions (including 7–8×10^8^ conidia/ml). The purpose of fracturing mycelia to a certain extent is to increase the efficiency of inoculation. The asexual mycelium is fractured to produce more progeny. The treatments consisted of 15 ml (A) fungal suspension, (B) extracellular metabolites produced by the test strain, (C) blank culture medium (PDB) and (D) sterile saline (control).

*P. massoniana* seeds collected from Guangzhou, Guangdong, China, were soaked in 1% potassium permanganate for 1 h and washed with running tap water for 3 h. After soaking in a petri dish at 20°C for 1 day, the seeds were wrapped with a piece of moist sterile cloth at 20°C in culture dishes for 1 day to accelerate germination. Geminated seeds were placed on pre-autoclaved (121°C, 2 h) soil in a container and covered with a 1–2 cm layer of autoclaved sand (121°C, 2 h). When the seedlings of Masson pine grew to 6–8 cm, we transplanted them with rhizosphere soil into a pot, one plant per pot (diameter=13 cm). Masson pine seedlings with similar shoot length and root crown diameter and with no sign of disease were cultured at 20°C and kept in good light and water conditions for later inoculation experiments. Each treatment group, including six inoculated Masson pine seedlings of the inoculation experiment, was applied on the soil surface of the root of Masson pine seedlings in the corresponding treatment group. The Masson pine seedlings were cultivated in yellow-brown soil from the arboretum of Nanjing Forestry University, China. The Masson pine seedlings were then transplanted in a greenhouse at 20°C. The shoot length and root crown diameter of the Masson pine seedlings were measured after culturing for 450 days. Every treatment included six biological replicates (six Masson pine seedlings) (*n*=6), each of which contained three technical replicates in each treatment (three data measurements).

### Identification of the efficient PSF strain

PSF isolated from Masson pine rhizosphere soil were cultured on Czapek yeast extract agar (CYA) (Kong, 2007) at 25°C for 1–2 weeks. The morphological characteristics of the colonies, mycelia, conidia and other fungal structures and physiological characteristics were recorded. The PSF strains were identified morphologically (Pitt, 1973; Christensen et al., 2000; Frisvad and Samson, 2004; Kong, 2007; Houbraken et al., 2011; Rivera and Seifert, 2011; Rivera et al., 2012). The morphologies of the conidiophores, conidiogenous cells, and conidia of the cultured strains were examined under a compound microscope (Axio Imager M2.0; Zeiss, Germany) equipped with a digital camera (AxioCam HRc, Zeiss, Germany) and in scanning electron microscopy (SEM; FEI Quanta 200, FEI, USA) specimens. The PSF strains were cultivated on PDA medium for 3 days and then rinsed three times with 2 ml 0.1 mol/l phosphate-buffered saline (PBS). The colonies were dehydrated in graded ethanol solutions and subjected to critical-point drying with liquid CO_2_ (EmiTech K850). Then the conidiophores and conidia were examined under SEM after sputtering with gold (Hitachi E-1010).

PSF were cultured on PDA medium at 25°C for 7 days. DNA was extracted and purified according to Ren et al. (2011). The *ben A* gene was amplified and sequenced with primers Bt2a and Bt2b; ITS with primers ITS1 and ITS4; *cox1* with primers PF and AR; *tef* with primers EF1c and EF6; and *cmd* with primers CMD5 and CMD6 (Rivera and Seifert, 2011). The PCR amplification consisted of 10 min at 95°C, followed by 35 cycles of 30 s at 95°C, 30 s at 56°C and 1 min at 72°C, with a final 5 min at 72°C. The PCR amplicons were sequenced at Beijing Huada Gene, China. All new sequences used for our analyses have been deposited in GenBank (Table S1).

### Phylogenetic analyses

The sequences that best matched each DNA gene sequence were selected after a blast search of the NCBI database (http://www.ncbi.nlm.nih.gov). The sequences were aligned using BioEdit software. A Jaccard dissimilarity matrix-based neighbor-joining phylogenetic tree with 1000 bootstrap replications was constructed separately for each of the alignments using MEGA (6.0) (El-Esawi et al., 2018). Tables S1 and S2 summarize the taxa, sequences and type materials. The type materials were elected based on Rivera and Seifert (2011).

### Statistical analyses

Microsoft Excel 2003 and SPSS (version 18.0, IBM, New York, USA) were used to collate and analyze the data of phosphate solubilization and Masson pine growth experiments. The data were expressed as mean±standard deviation (s.d.). One-way ANOVA Duncan's multiple range test (two-tailed) was used to perform significant differences and multiple comparison analyses (no adjustment) of phytase and phosphatase activity, solubilization of different inorganic insoluble phosphates and phosphate-solubilizing rate by JP-NJ2 as well as the shoot length and root crown diameter of Masson pine seedlings. Statistical difference of phytase and phosphatase activity was determined at *P*<0.01; for solubilization of different inorganic insoluble phosphates, and for phosphate-solubilizing rate by JP-NJ2, as well as the shoot length and root crown diameter of Masson pine seedlings, statistical difference was determined at *P*<0.05.

## Supplementary Material

Supplementary information
