# Supplementary material for: The phosphate-solubilizing ability of Penicillium guanacastense and its effects on the growth of Pinus massoniana in phosphate-limiting conditions
Source: Biol Open. 2019 Nov 8;8(11):bio046797. doi: 10.1242/bio.046797 (PMC6899000; doi:10.1242/bio.046797)
Supplement: Supplementary information [file biolopen-8-046797-s1.pdf]

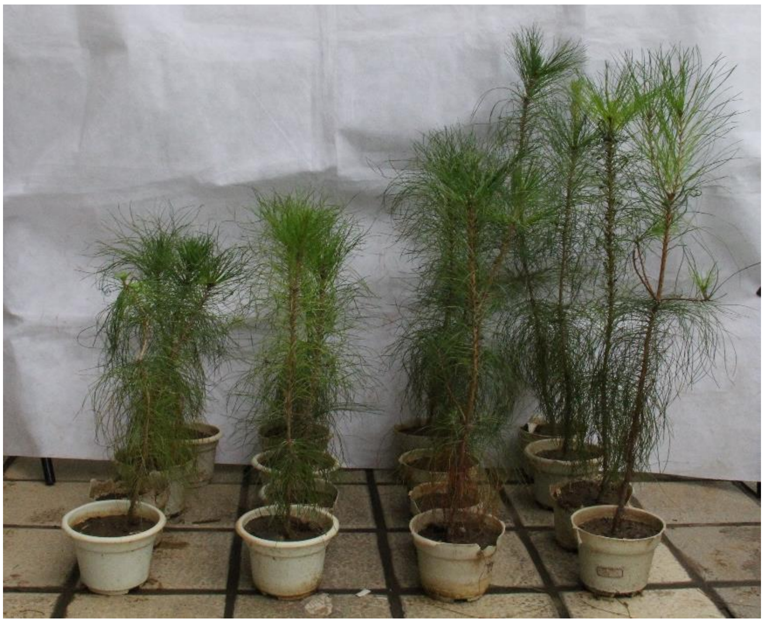

D C B A

Fig. S1 Growth-promoting ability of highly efficient phosphate-solubilizing fungus JP-NJ2 on *p. massoniana* after culturing for 450 days. Inoculation test was divided into four treatments, each of which was treated with 15mL of corresponding substance. A) fungal suspension; B) extracellular metabolites produced by the test strain; C) blank culture medium (PDB); D) sterile saline (control). Each treatment was repeated 20 times, and the inoculated pine seedlings were placed in a greenhouse (20°C), with unified management and timely watering. Every treatment included six biological replicates (six masson pine seedlings) (n=6), each of which contained three technical replicates in each treatment (three data measurements). (Only four of the six plants are shown in figure).

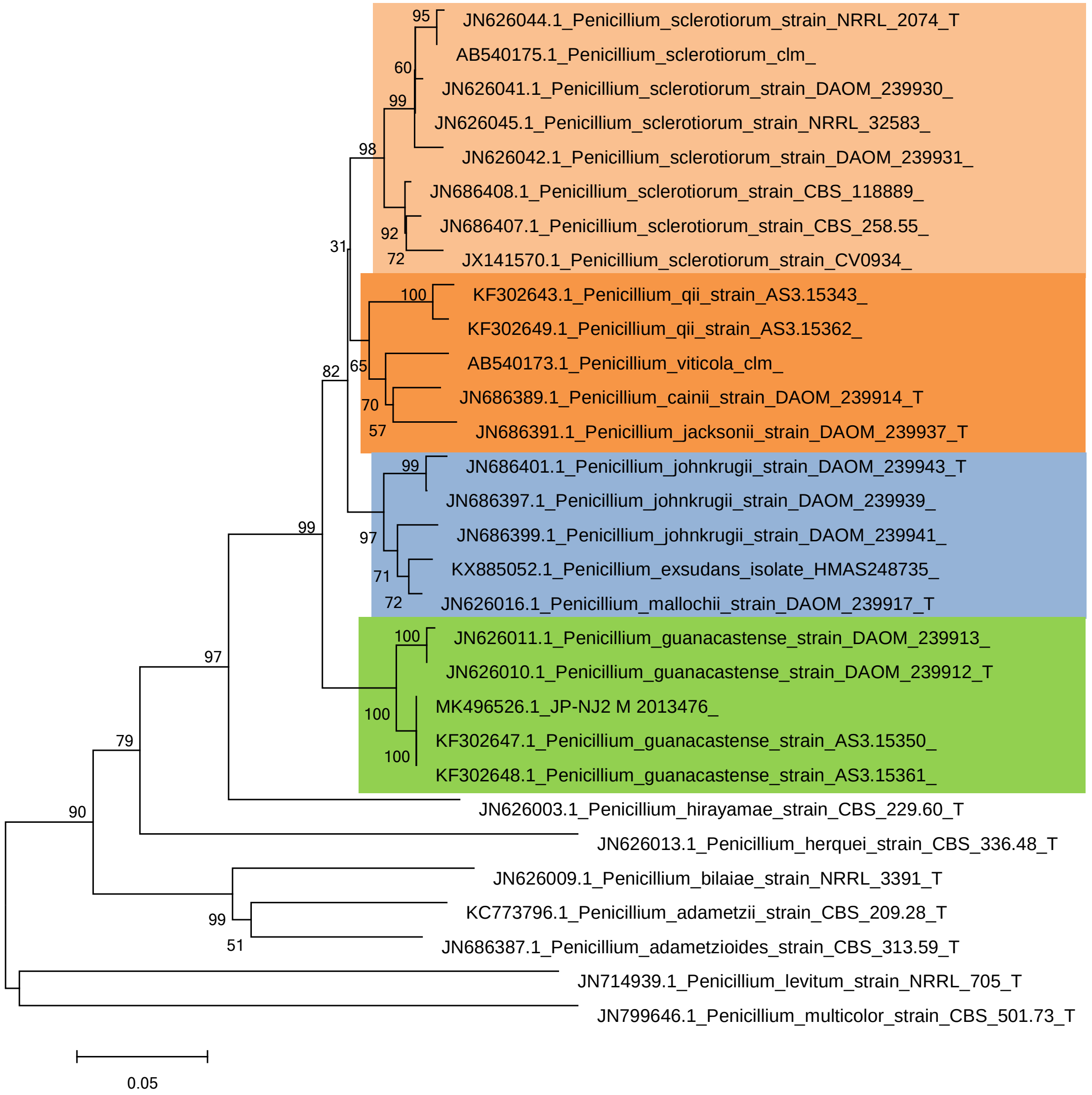

Fig. S2 Neighbor-joining tree of phosphate-solubilizing fungus JP-NJ2 based on *cmd* sequences. Bootstrap values on 1000 replications are shown at nodes of the tree. Scale bar was 0.05substitutions per nucleotide position. T indicates ex type.

Biology Open • Supplementary information

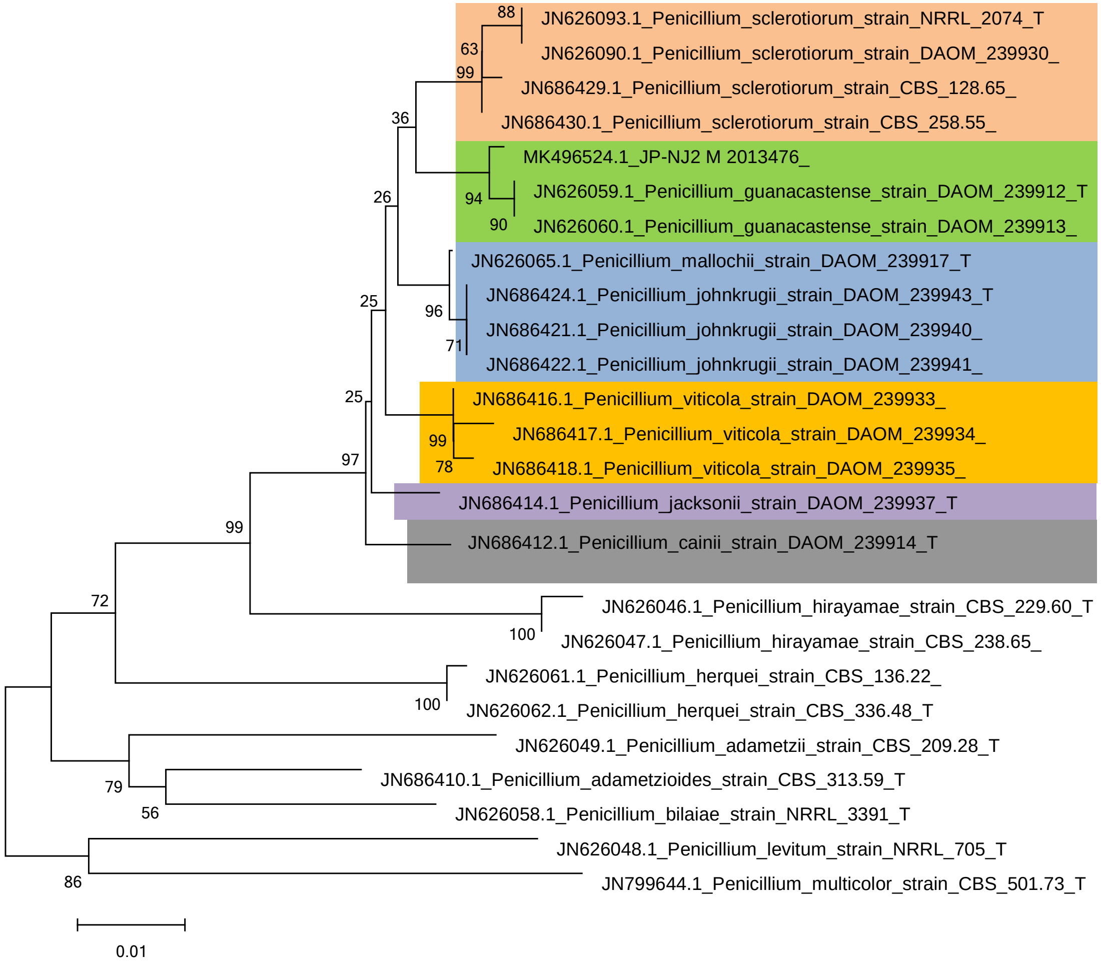

Fig. S3 Neighbor-joining tree of phosphate-solubilizing fungus JP-NJ2 based on *cox1* sequences. Bootstrap values on 1000 replications are shown at nodes of the tree. Scale bar was 0.01 substitutions per nucleotide position. T indicates ex type.

Biology Open • Supplementary information

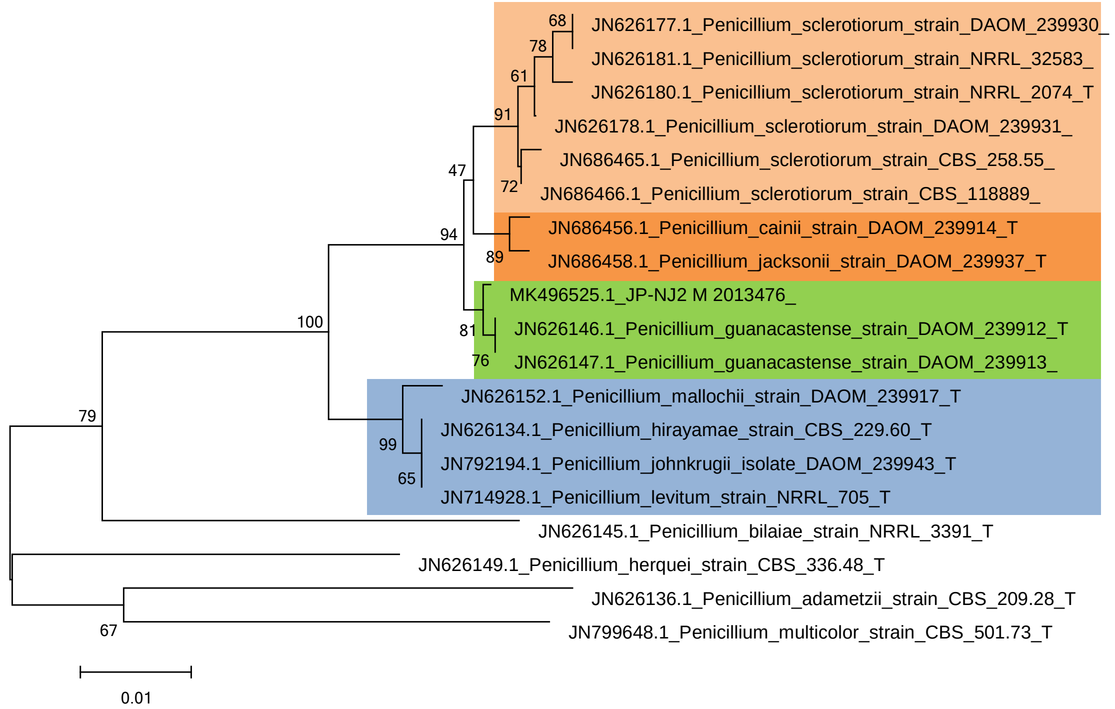

Fig. S4 Neighbor-joining tree of phosphate-solubilizing fungus JP-NJ2 based on *tef* sequences. Bootstrap values on 1000 replications are shown at nodes of the tree. Scale bar was 0.01 substitutions per nucleotide position. T indicates ex type.

Biology Open • Supplementary information

Table S1: Accession numbers of materials, isolation details and GenBank accession numbers for the five genes/region used for phylogenetic analysis of the phosphate-solubilizing fungus JP-NJ2.

Materials	Accession number	Location	Host or substrate	GenBank Accession number				
				ITS	benA	tef	cmd	cox1
**JP-NJ2**	M 2013476	China, Nanjing	Rhizosphere soil from ***Pinus massoniana***	KF991208	MK496527	MK496525	MK496526	MK496524
Penicillium guanacastense	DAOM 239912 (T)	Costa Rica, Santa Rosa, Área de Conservación Guanacaste	Gut of the caterpilar ***Eutelia*** sp. reared on leaves of ***Spondias mombin***	NR_111673	JN625967	JN626146	JN626010	JN626059
Penicillium guanacastense	DAOM 239913	Costa Rica, Santa Rosa, Área de Conservación Guanacaste	Gut of the caterpilar ***Eutelia*** sp. reared on leaves of ***Spondias mombin***	JN626099	JN625968	JN626147	JN626011	JN626060
Penicillium guanacastense	AS3.15350	China	Unknown	KF302657	KF302637	-	KF302647	-
Penicillium guanacastense	AS3.15361	China	Unknown	KF302658	KF302638	-	KF302648	-
Penicillium guanacastense	DLM114	South Korea	Root of coastal plant	KU555971	-	-	-	-
Penicillium guanacastense	ELM148	South Korea	Root of coastal plant	KU556085	-	-	-	-
Penicillium	NW_11	South Africa	Cashew nut	MG576102	-	-	-	-

Biology Open • Supplementary information

guanacastense								
Penicillium guanacastense	WSO111	South Korea	Root of coastal plant	KU556125	-	-	-	-
Penicillium guanacastense	XQ22	China	***Euphausia superba***	KU216724	-	-	-	-
Penicillium cainii	CNU 114236	Unknown	Unknown	-	KC424616	-	-	-
Penicillium cainii	DAOM 239914 (T)	Canada: Ontario, Niagara, Niagara Falls, Fireman's Park	Soil, forest rich in Juglandaceae	-	JN686366	-	-	JN686412
Penicillium cainii	DTO312-G5	Canada	House dust	-	MF803842	-	-	-
Penicillium daejeonium	SFC101564	South Korea	Egg mass of ***Arctoscopus japonicus***	-	MF176798	-	-	-
Penicillium daejeonium	SFC 20160805M22	Unknown	Unknown	-	KX712486	-	-	-
Penicillium daejeonium	SFC 20160805M23	Unknown	Unknown	-	KX712487	-	-	-
Penicillium exsudans	HMAS248735	Unknown	Unknown	-	KX885042	-	KX885052	-
Penicillium hirayamae	CBS 229.60	Thailand	Milled rice	-	-	-	-	JN626046
Penicillium hirayamae	CBS 238.65	South Africa	Corn meal	-	-	-	-	JN626047
Penicillium jacksonii	DAOM 239937 (T)	Australia, Queensland, Barrine Lake	Forest soil	NR_111675	JN686368	JN686458	JN686391	JN686414
Penicillium jacksonii	DAOM 239938	Australia: Queensland, Barrie Lake	Forest soil	JN686438	-	-	-	-

Biology Open • Supplementary information

Penicillium johnkrugii	DAOM 239939	Malaysia: Kedah, Langkawi	Rainforest soil	-	-	-	JN686397	-
Penicillium johnkrugii	DAOM 239940	Malaysia: Kedah, Langkawi	Forest soil	-	-	-	-	JN686421
Penicillium johnkrugii	DAOM 239941	Malaysia: Kedah, Langkawi	Forest soil	-	--	--	JN686399	JN686422
Penicillium johnkrugii	DAOM 239946	Malaysia: Kedah, Langkawi	Forest soil	JN686450	-	-	-	-
Penicillium mallochii	CCDCA 10735	Unknown	***Himatanthus* sp**	-	KY508648	-	-	-
Penicillium mallochii	DAOM 239917	Costa Rica: Santa Rosa, Area de Conservacion	***Spondias mombin***	-	JN625973	-	-	JN626065
Penicillium mallochii	DAOM 239918	Costa Rica: Santa Rosa, Area de Conservacion Guanacaste	***Rothschildia lebeau***	-	JN625974	-	-	-
Penicillium mallochii	DAOM 239922	Costa Rica, Santa Rosa, Área de Conservación Guanacaste	***Rothschildia lebeau***	JN626109	-	-	-	-
Penicillium mallochii	SA2I6F1	Unknown	Curculionidae sp		KX421475			
Penicillium multicolor	CBS 128423	USA: Wisconsin	Unknown	MH864923	-	-	-	-
Penicillium	AS3.15340	Unknown	Unknown	--	KF302631	-	-	-

Biology Open • Supplementary information

qii								
Penicillium qii	AS3.15343	Unknown	Unknown	-	KF302633	-	KF302643	-
Penicillium qii	AS3.15348	Unknown	Unknown	-	KF302636	-	-	-
Penicillium qii	AS3.15362	Unknown	Unknown	-	KF302639	-	KF302649	-
Penicillium sclerotiorum	A13	Unknown	Unknown	EF564151	-	-	-	-
Penicillium sclerotiorum	BT2	Indonesia	Air	-	AB540176	-	-	-
Penicillium sclerotiorum	CBS 118889	South Korea	Unknown	-	JN686385	JN686466	JN686408	-
Penicillium sclerotiorum	clm	Indonesia	Air	-	-	-	AB540175	-
Penicillium sclerotiorum	NRRL 32583	USA: Hawaii, Kuaunai	Coffee	-	JN626002	JN626181	JN626045	-
Penicillium sclerotiorum	DAOM 239930	Unknown	Pineapple soil	-	JN625998	JN626177	JN626041	JN626090
Penicillium sclerotiorum	CBS 128.65	Zaire: Leopodville, Nsang-Ngidin ga River	Forest soil	-	-	-	-	JN686429
Penicillium sclerotiorum	CBS 258.55	Turkey: Istanbul	Culture contaminant	JN686453	JN686384	JN686465	JN686407	JN686430
Penicillium sclerotiorum	DAOM 239931	Australia: Queensland, Barron Falls	Forest soil	-	JN625999	JN626178	JN626042	-
Penicillium sclerotiorum	DTO 090-I7	Unknown	Unknown	-	KM088790	-	-	-
Penicillium sclerotiorum	CV0934	Unknown	Unknown	-	-	-	JX141570	-
Penicillium	NRRL	Indonesia:	Air	JN626132	JN626001	JN626180	JN626044	-

sclerotiorum	2074(T)	Java, Buitenzorg						
Penicillium sclerotiorum	IFO 6105	Unknown	Unknown	-	LC057670	-	-	-
Penicillium vanoranjei	DTO119G8	Unknown	Unknown	KC695692	KC695682	-	-	-
Penicillium vanoranjei	DTO99F3	Unknown	Unknown	-	KC695684	-	-	-
Penicillium vanoranjei	CBS 134406 (T)	Unknown	Unknown	NR_120267	-	-	-	-
Penicillium viticola	OUCMB I110151	China: Qingdao	***Ahnfeltiopsis flabelliformis***	JX003128	-	-	-	-
Penicillium viticola	OUCMB I110152	China: Qingdao	***Ahnfeltiopsis flabelliformis***	JX003129	-	-	-	-
Penicillium viticola	DAOM 239933	Australia: Queensland, Baron Falls	Forest soil	-	JN686370	-	-	JN686416
Penicillium viticola	DAOM 239934	Australia: Queensland, Atherton	Forest soil	-	JN686371	-	-	JN686417
Penicillium viticola	DAOM 239935	Australia: Queensland, Atherton	Rainforest soil	-	JN686372	-	-	JN686418
Penicillium viticola	clm	Japan	Grape	-	-	-	AB540173	-

The selected accession numbers of materials are based on the result of NCBI standard nucleotide blast, which provides sequences producing significant alignments. All accession numbers of materials from *Penicillium guanacastense* are considered to generate the phylogenetic tree. Genus and species in the columns are represented by bold Italic. T indicates ex type.

Table S2: Accession numbers of ex type materials, isolation details and GenBank accession numbers for the five genes/region used for phylogenetic analysis of the JP-NJ2.

Materials	Accession number	Location	Host or substrate	GenBank Accession number				
				ITS	benA	tef	cmd	cox1
Penicillium adametzii	CBS 209.28 (T)	Poland, Poznan	Soil under conifers	NR_103661	JN625957	JN626136	KC773796	JN626049
Penicillium adametzioides	CBS 313.59 (T)	Japan	Soil	NR_103660	JN799642	-	JN686387	JN686410
Penicillium bilaiae	NRRL 3391 (T)	Ukraine, Kiev	Soil	NR_111679	JN625966	JN626145	JN626009	JN626058
Penicillium cainii	DAOM 239914 (T)	Canada, Ontario, Niagara, Niagara Falls, Fireman's Park	Soil, forest rich in Juglandaceae	NR_120000	JN686366	JN686456	JN686389	JN686412
Penicillium guanacastense	DAOM 239912 (T)	Costa Rica, Santa Rosa, Área de Conservación Guanacaste	Gut of the caterpilar ***Eutelia*** sp. reared on leaves of ***Spondias mombin***	NR_111673	JN625967	JN626146	JN626010	JN626059
Penicillium herquei	CBS 336.48 (T)	France	Leaf of ***Agauria pirifolia***	NR_103659	JN625970	JN626149	JN626013	JN626062
Penicillium hirayamae	CBS 229.60 (T)	Thailand	Milled rice	NR_111672	JN625955	JN626134	JN626003	JN626046
Penicillium jacksonii	DAOM 239937 (T)	Australia, Queensland, Barrine Lake	Forest soil	NR_111675	JN686368	JN686458	JN686391	JN686414
Penicillium johnkrugii	DAOM 239943 (T)	Malaysia, Kedah,	Rainforest soil	NR_111676	JN686378	JN792194	JN686401	JN686424

Biology Open • Supplementary information

		Langkawi, Gunung Raya Rainforest						
Penicillium levitum	NRRL 705 (T)	USA, New York	Modeling clay	JN626097	JN714938	JN714928	JN714939	JN626048
Penicillium mallochii	DAOM 239917 (T)	Costa Rica, Santa Rosa, Área de Conservación Guanacaste	Caterpillar on ***Spondias mombin***	NR_111674	JN625973	JN626152	JN626016	JN626065
Penicillium multicolor	CBS 501.73 (T)	Russia	Soil	NR_111870	JN799645	JN799648	JN799646	JN799644
Penicillium sclerotiorum	NRRL 2074 (T)	Indonesia, Java, Buitenzorg	Air	JN626132	JN626001	JN626180	JN626044	JN626093
Penicillium viticola	JCM 17636 (T)	Japan , Yamanashi	Grape	NR_121209	-	-	-	-

The result of NCBI standard nucleotide blast is considered preferentially, then the aim of type materials genus *Penicillium* added is to make the phylogenetic tree more plentiful. Genus and species in the columns are represented by bold Italic. T indicates ex type.

Biology Open • Supplementary information
